# Supplementary material for: The Role of Co-Deleted Genes in Neurofibromatosis Type 1 Microdeletions: An Evolutive Approach
Source: Genes (Basel). 2019 Oct 24;10(11):839. doi: 10.3390/genes10110839 (PMC6896060; doi:10.3390/genes10110839)
Supplement: Supplementary file 1 [file genes-10-00839-s001.pdf]

Table S1. Clinical characterization of the NF1 patients previously analyzed.

Patient	Age at onset/gender	Age	Familial	Mutation	Riccardi/Ablon	Main symptoms	Other pathologies/characteristics
1	At birth/female	26	No	Type I *NF1* microdeletion	4/moderate	CALM, cutaneous and malignant peripheral nerve sheath tumour, axillary and inguinal freckling, Lisch nodules, escoliosis, cognitive deficit	No
2	4 years/male	57	Yes	Atypical *NF1* microdeletion	2/moderate	CALM, cutaneous neurofibromas, axillary and inguinal freckling	Hemocromatosis, hipogonadism
3	4 years/male	46	Yes	Atypical *NF1* microdeletion	2/mild	CALM, cutaneous and plexiform neurofibromas, axillary and inguinal freckling, macrocephaly	No
4	4 years/male	14	No	Atypical *NF1* microdeletion	2/moderate	CALM, cutaneous neurofibromas, axillary and inguinal freckling, macrocephaly	Dysmorphic features, oblique palpebral fissures, protruding ears and tall stature

CALM = Café-au-lait macules.

Table S2. Information about the sequences obtained in the Ensembl and NCBI databases for all species included in this study.

Specie	Gene	Protein code	Locus name	Gene	Number of transcripts	Genomic location	Score	E-value	%ID
Cattle (Bos taurus - Bta) 60 chromosomes (29+X)	*CRLF3*	ENSBTAP00000024457	ENSBTAG00000018381	*CRLF3*	1	19:18456446-18498690	642	0.0	95.66
	ATAD5	ENSBTAP00000024464	ENSBTAG00000018383	*ATAD5*	1	19:18411252-18445391	1955	0.0	76.77
	ADAP2/	ENSBTAP00000004328	ENSBTAG00000003334	*ADAP2*	1	19:18354230-18388232	517	0.0	87.21
	RNF135	ENSBTAP00000022890	ENSBTAG00000017225	*RNF135*	1	19:18331445-18343178	412	1,00E-153	64.60
	NF1	ENSBTAP00000015699	ENSBTAG00000011829	*NF1*	2	19:18973027-19183670	4131	0.0	97.17
	UTP6	ENSBTAP00000020740	ENSBTAG00000015612	*UTP6*	1	19:18570929-18596526	703	0.0	74.75
	SUZ12	ENSBTAP00000024971	ENSBTAG00000018752	*SUZ12*	1	19:18507875-18544756	1031	0.0	99.24
	OMG	ENSBTAP00000035220	ENSBTAG00000025213	*OMG*	1	19:19045123-19046406	571	0.0	95.09
	LRRC37B	ENSBTAP00000004849	ENSBTAG00000027274	*ENSBTAG00000027274*	2	19:45804030-45845485	424	7,00E-147	42.35
	EVI2A	ENSBTAP00000012311	ENSBTAG00000009354	*EVI2A*	1	19:19023409-19024104	279	1,00E-101	79.74
	EVI2B	ENSBTAP00000012309	ENSBTAG00000009353	*EVI2B*	1	19:19034875-19036206	407	2,00E-151	67.19
	RAB11FIP4	ENSBTAP00000022573	ENSBTAG00000016972	*RAB11FIP4*	1	19:18851512-18886471	673	0.0	94.11
	TEFM	ENSBTAP00000024467	ENSBTAG00000018386	*C19H17orf42*	1	19:18400772-18407786	420	1,00E-157	76.55
	COPRS	ENSBTAP00000002453	ENSBTAG00000001885	*COPRS*	1	19:18604745-18608931	219	8,00E-78	76.88
Marmoset (Callithrix jacchus - Cja) 48 chromosomes	*CRLF3*	ENSCJAP00000026067	ENSCJAG00000014163	*CRLF3*	3	5:92519493-92566475	653	0.0	98.19
	ATAD5	ENSCJAP00000026098	ENSCJAG00000014179	*ATAD5*	1	5:92433480-92513351	2253	0.0	87.82
	ADAP2	ENSCJAP00000026172	ENSCJAG00000014210	*ADAP2*	1	5:92369119-92406702	554	0.0	93.44
	RNF135	ENSCJAP00000026182	ENSCJAG00000014235	*RNF135*	3	5:92342252-92357613	560	0.0	85.75

(22+X+Y)	*NF1*	ENSCJAP00000025805	ENSCJAG00000013924	*NF1*	6	5:93187983-93481033	4293	0.0	99.68
	UTP6	ENSCJAP00000025966	ENSCJAG00000014115	*UTP6*	1	5:92698514-92738377	866	0.0	95.64
	SUZ12	ENSCJAP00000026016	ENSCJAG00000014133	*SUZ12*	1	5:92580829-92648466	1035	0.0	99.39
	OMG	ENSCJAP00000025889	ENSCJAG00000014092	*OMG*	1	5:93269084-93270367	592	0.0	97.90
	LRRC37B	ENSCJAP00000044215	ENSCJAG00000001032	*ENSCJAG00000001032*	3	GL285097.1:40089-55767	541	0.0	53.95
		ENSCJAP00000026099	ENSCJAG00000014194	*ENSCJAG00000014194*	1	13:20088200-20090218	259	4,00E-86	41.26
		ENSCJAP00000002355	ENSCJAG00000001338	*ENSCJAG00000001338*	3	5:63796051-63811610	227	6,00E-73	71.35
		ENSCJAP00000032794	ENSCJAG00000017781	*ENSCJAG00000017781*	1	5:38440670-38443673	209	4,00E-66	41.30
	EVI2A	ENSCJAP00000025894	ENSCJAG00000014096	*ENSCJAG00000014096*	1	5:93241274-93243377	365	3,00E-136	91.02
	EVI2B	ENSCJAP00000046393	ENSCJAG00000014095	*EVI2B*	2	5:93256893-93258209	536	0.0	87.70
	RAB11FIP4	XP_002748389	Gene ID: 100393768	*RAB11FIP4*	2	5:NC_013900.1	878	0.0	99.00
	TEFM	ENSCJAP00000026127	ENSCJAG00000014201	*TEFM*	1	5:92423632-92429548	481	0.0	87.64
	COPRS	XP_008995376	Gene ID: 100391809	*COPRS*	3	5:NC_013900.1	302	2,00E-104	88.00
Dog (Canis lupus familiaris - Clu) 78 chromosomes (38+X)	*CRLF3*	ENSCAFP00000027303	ENSCAFG00000018492	*CRLF3*	1	9:NC_006591.3	823	0.0	96.00
	ATAD5	ENSCAFP00000027299	ENSCAFG00000018481	*ATAD5*	2	9:40870927-40917915	1973	0.0	76.70
	ADAP2	ENSCAFP00000030645	ENSCAFG00000018466	*ADAP2*	1	9:40819428-40845115	532	0.0	89.24
	RNF135	XP_005624884	Gene ID: 480614	*RNF135*	1	9:NC_006591.3	535	0.0	65.00
	NF1	ENSCAFP00000027461	ENSCAFG00000018592	*NF1*	2	9:41470995-41679382	4225	0.0	98.51
	UTP6	ENSCAFP00000027330	ENSCAFG00000018507	*UTP6*	1	9:41067262-41093010	809	0.0	89.46
	SUZ12	ENSCAFP00000027313	ENSCAFG00000018498	*SUZ12*	1	9:40994870-41038250	1020	0.0	99.23
	OMG	ENSCAFP00000027481	ENSCAFG00000018624	*OMG*	1	9:41546736-41548019	583	0.0	96.26
	*LRRC37B**	ENSCAFP00000041156	ENSCAFG00000031375	*ENSCAFG00000031375*	1	9:17979631-17995435	286	1,00E-93	38.47
		ENSCAFP00000037618	ENSCAFG00000029478	*ENSCAFG00000029478*	1	9:10291948-10304722	246	6,00E-75	36.31
		ENSCAFP00000043093	ENSCAFG00000031467	*ENSCAFG00000031467*	1	14:22322624-22332292	158	2,00E-44	30.58
	EVI2A	ENSCAFP00000036800	ENSCAFG00000029405	*ENSCAFG00000029405*	1	9:41523246-41523938	260	1,00E-93	74.03
	EVI2B	ENSCAFP00000027479	ENSCAFG00000018623	*EVI2B*	1	9:41535738-41537066	447	2,00E-167	72.52
	RAB11FIP4	ENSCAFP00000027341	ENSCAFG00000018522	*RAB11FIP4*	1	9:41342258-41416095	719	0.0	94.25
	TEFM	ENSCAFP00000042698	ENSCAFG00000018475	*TEFM*	2	9:40860963-40867816	429	2,00E-161	76.11
	COPRS	ENSCAFP00000027336	ENSCAFG00000018517	*COPRS*	1	9:41100041-41103771	192	9,00E-67	81.62
Gorilla (Gorilla gorilla gorilla - Ggo) 46 chromosomes (22+X) chromosomes 2 divided in 2a e 2b)	*CRLF3*	XP_004042017	Gene ID: 101150086	*CRLF3*	1	5:NC_018429.1	878	0.0	100.00
	ATAD5	ENSGGOP00000005765	ENSGGOG00000005883	*ATAD5*	1	5:53366215-53428754	2363	0.0	93.71
	ADAP2	XP_004042029	Gene ID: 101126041	*ADAP2*	2	5:53303421-53338277	668	0.0	89.00
	RNF135	XP_004042030	Gene ID: 101126404	*RNF135*	1	5:NC_018429.1	679	0.0	98.00
	NF1	ENSGGOP00000006859	ENSGGOG00000006983	*NF1*	2	5:52834636-53161679	4209	0.0	98.20
	UTP6	ENSGGOP00000028108	ENSGGOG00000026003	*ENSGGOG00000026003*	2	15:30768738-30770828	671	0.0	78.76
		ENSGGOP00000013242	ENSGGOG00000013569	*UTP6*	1	5:52298783-52338080	611	0.0	74.20

	SUZ12	ENSGGOP00000010105	ENSGGOG00000010352	*SUZ12*	1	5:52191094-52251152	920	0.0	99.66
	OMG	ENSGGOP00000046509	ENSGGOG00000039026	*OMG*	1	CABD030155035.1:2722-4005	790	0.0	98.83
	LRRC37B	ENSGGOP00000024608	ENSGGOG00000012882	*ENSGGOG00000012882*	2	10:30462810-30468162	977	0.0	79.89
		ENSGGOP00000028210	ENSGGOG00000026947	*ENSGGOG00000026947*	1	5:52148708-52163228	784	0.0	83.88
		ENSGGOP00000024093	ENSGGOG00000025742	*ENSGGOG00000025742*	1	5:53207699-53219434	413	6,00E-147	51.62
		ENSGGOP00000027053	ENSGGOG00000024250	*ENSGGOG00000024250*	1	5:45127944-45141094	354	1,00E-122	53.10
	EVI2A	ENSGGOP00000025097	ENSGGOG00000024807	*ENSGGOG00000024807*	2	5:52889964-52892080	399	9,00E-151	98.46
	EVI2B	ENSGGOP00000005071	ENSGGOG00000005182	*EVI2B*	1	5:52905461-52906765	614	0.0	97.93
	RAB11FIP4	ENSGGOP00000001056	ENSGGOG00000001071	*RAB11FIP4*	1	5:52672926-52816615	869	0.0	99.68
	TEFM	ENSGGOP00000020628	ENSGGOG00000025274	*TEFM*	1	5:53354081-53361907	554	0.0	99.44
	COPRS	ENSGGOP00000007136	ENSGGOG00000007292	*COPRS*	2	5:52342244-52349288	281	4,00E-103	97.31
Human (homo sapiens - Hsa) 48 chromosomes (46+X+Y)	*CRLF3*	ENSP00000318804	ENSG00000176390	*CRLF3*	1	17:30784190-30824651	667	0.0	100.00
	ATAD5	ENSP00000313171	ENSG00000176208	*ATAD5*	2	17:30832348-30894910	2574	0.0	100.00
	ADAP2	ENSP00000329468	ENSG00000184060	*ADAP2*	6	17:30922015-30957866	591	0.0	100.00
	RNF135	ENSP00000328340	ENSG00000181481	*RNF135*	5	17:30971074-30999188	655	0.0	100.00
	NF1	ENSP00000351015	ENSG00000196712	*NF1*	7	17:31095310-31374152	4302	0.0	100.00
	UTP6	ENSP00000261708	ENSG00000108651	*UTP6*	1	17:31863362-31901627	908	0.0	100.00
	SUZ12	ENSP00000316578	ENSG00000178691	*SUZ12*	2	17:31937448-31998958	1040	0.0	100.00
	OMG	ENSP00000247271	ENSG00000126861	*OMG*	1	17:31295048-31296331	609	0.0	100.00
	LRRC37B	ENSP00000378202	ENSG00000185158	*LRRC37B*	9	17:32021147-32053325	1295	0.0	100.00
	EVI2A	ENSP00000247270	ENSG00000126860	*EVI2A*	5	17:31318306-31320431	404	2,00E-152	100.00
	EVI2B	ENSP00000462682	ENSG00000185862	*EVI2B*	2	17:31304305-31305609	625	0.0	100.00
	RAB11FIP4	ENSP00000482620	ENSG00000131242	*RAB11FIP4*	4	17:31391892-31531729	870	0.0	100.00
	TEFM	ENSP00000462963	ENSG00000172171	*TEFM*	3	17:30899172-30906198	557	0.0	100.00
	COPRS	ENSP00000304327	ENSG00000172301	*COPRS*	3	17:31852142-31859199	283	9,00E-104	98.39
Macaque (Macaca mulatta - Mmu) 42 chromosomes (20+X)	*CRLF3*	ENSMMUP00000011900	ENSMMUG00000009069	*CRLF3*	1	16:27225529-27279056	444	0.0	99.10
		ENSMMUP00000021240	ENSMMUG00000016154	*ENSMMUG00000016154*	1	6:96081251-96082594	420	4,00E-157	68.49
	ATAD5	ENSMMUP00000050281.1	ENSMMUG00000040177	*ATAD5*	1	16:24784775-24786671	3204	0.0	96.27
	ADAP2	ENSMMUP00000011908	ENSMMUG00000009077	*ADAP2*	1	16:27378497-27417323	572	0.0	96.06
	RNF135	XP_014974482	Gene ID: 713821	*RNF135*	1	16:NC_027908.1	758	0.0	90.00
	NF1	XP_014974491	Gene ID: 712911	*NF1*	3	16:NC_027908.1	5816	0.0	99.00
	UTP6	ENSMMUP00000026846	ENSMMUG00000020403	*UTP6*	2	16:27056346-27097385	881	0.0	97.49
	SUZ12	XP_014974503	Gene ID: 717834	*SUZ12*	1	16:NC_027908.1	1382	0.0	99.00
	OMG	ENSMMUP00000008966	ENSMMUG00000006824	*OMG*	1	16:26468665-26469948	594	0.0	98.36
	LRRC37B	ENSMMUP00000024288	ENSMMUG00000018463	*ENSMMUG00000018463*	3	16:56955303-56992180	975	0.0	77.75
		ENSMMUP00000015398	ENSMMUG00000011880	*ENSMMUG00000011880*	5	16:57039966-57077828	622	0.0	79.90

		ENSMMUP00000004409	ENSMMUG00000003308	*ENSMMUG00000003308*	2	16:55751910-55764041	288	2,00E-97	83.33
		ENSMMUP00000004282	ENSMMUG00000011737	*ENSMMUG00000011737*	2	16:54000170-54002322	273	9,00E-91	47.50
	EVI2A	ENSMMUP00000035028	ENSMMUG00000008604	*ENSMMUG00000008604*	2	16:26495866-26497991	387	9,00E-146	94.98
	EVI2B	ENSMMUP00000011283	ENSMMUG00000008603	*EVI2B*	1	16:26481610-26482923	566	0.0	90.64
	RAB11FIP4	ENSMMUP00000010642	ENSMMUG00000008127	*RAB11FIP4*	2	16:26609307-26711478	760	0.0	99.46
	TEFM	ENSMMUP00000011905	ENSMMUG00000009073	*TEFM*	1	16:27355262-27361704	519	0.0	93.63
	COPRS	XP_001112181	Gene ID: 713293	*COPRS*	2	16:NC_027908.1	338	3,00E-118	95.00
Mice (Mus musculus - Mus) 42 chromosomes (19+X+Y)	*CRLF3*	ENSMUSP00000060028	ENSMUSG00000017561	*Crlf3*	3	11:NC_000077.6	631	0.0	92.99
	ATAD5	ENSMUSP00000103874	ENSMUSG00000017550	*Atad5*	2	11:80089771-80134368	1670	0.0	67.82
	ADAP2	ENSMUSP00000021050	ENSMUSG00000020709	*Adap2*	2	11:80154216-80178415	514	0.0	85.41
	RNF135	ENSMUSP00000017839	ENSMUSG00000020707	*Rnf135*	1	11:80183917-80199084	369	1,00E-135	57.70
	NF1	ENSMUSP00000071289	ENSMUSG00000020716	*Nf1*	3	11:79340000-79578393	4247	0.0	98.42
	UTP6	ENSMUSP00000046643	ENSMUSG00000035575	*Utp6*	2	11:79935958-79962309	785	0.0	86.10
	SUZ12	ENSMUSP00000017692	ENSMUSG00000017548	*Suz12*	2	11:79993527-80032145	1021	0.0	97.87
	OMG	ENSMUSP00000132918	ENSMUSG00000049612	*Omg*	1	11:79501741-79503021	542	0.0	89.23
	LRRC37B	ENSMUSP00000129662	ENSMUSG00000034239	*Gm884*	2	11:103543324-103614303	329	2,00E-109	38.29
	EVI2A	ENSMUSP00000125936	ENSMUSG00000078771	*Evi2a*	2	11:79527117-79527782	210	6,00E-73	64.04
	EVI2B	ENSMUSP00000128569	ENSMUSG00000070354	*Evi2b*	1	11:79515470-79516747	307	7,00E-110	53.39
	RAB11FIP4	ENSMUSP00000017783	ENSMUSG00000017639	*Rab11fip4*	2	11:79591315-79692836	793	0.0	92.64
	TEFM	ENSMUSP00000059304	ENSMUSG00000046909	*Tefm*	2	11:80136848-80142077	361	2,00E-133	67.90
	COPRS	ENSMUSP00000033839	ENSMUSG00000031458	*Coprs*	1	8:13885035-13890211	204	1,00E-71	75.74
Gibon (Nomascus leucogenys - Nle) 38-52 chromosomes	*CRLF3*	XP_003279430	Gene ID: 100599101	*CRLF3*	1	14: NC_019829.1	683	0.0	100.00
	ATAD5	ENSNLEP00000007107	ENSNLEG00000005853	*ATAD5*	1	GL397459.1:87678-148528	2474	0.0	96.31
	ADAP2	XP_012359120	Gene ID: 100599439	*ADAP2*	1	14: NC_019829.1	655	0.0	89.00
	RNF135	----------	----------	----------	----------	----------	-	-	-
	NF1	ENSNLEP00000013316	ENSNLEG00000010582	*NF1*	1	GL397485.1:116905-400800	4277	0.0	99.08
	UTP6	ENSNLEP00000013490	ENSNLEG00000011009	*UTP6*	1	GL397485.1:907327-950700	875	0.0	97.15
	SUZ12	ENSNLEP00000013539	ENSNLEG00000011117	*SUZ12*	1	GL397485.1:982109-1055613	1035	0.0	99.54
	OMG	ENSNLEP00000013331	ENSNLEG00000010979	*OMG*	1	GL397485.1:322932-324215	598	0.0	98.13
	LRRC37B	ENSNLEP00000013553	ENSNLEG00000011154	*LRRC37B*	1	GL397485.1:1078267-1111947	1106	0.0	85.19
		ENSNLEP00000005752	ENSNLEG00000004744	*ENSNLEG00000004744*	1	GL397331.1:9257466-9291479	573	0.0	66.78
	EVI2A	ENSNLEP00000013338	ENSNLEG00000010986	*ENSNLEG00000010986*	1	GL397485.1:346257-348375	384	2,00E-144	94.98
	EVI2B	ENSNLEP00000013335	ENSNLEG00000010983	*EVI2B*	1	GL397485.1:332211-333518	598	0.0	95.18
	RAB11FIP4	ENSNLEP00000013346	ENSNLEG00000010989	*RAB11FIP4*	2	GL397485.1:435495-560387	800	0.0	92.28
	TEFM	ENSNLEP00000007111	ENSNLEG00000005875	*TEFM*	1	GL397459.1:152769-160546	536	0.0	97.20
	COPRS	ENSNLEP00000013362	ENSNLEG00000011000	*COPRS*	1	GL397485.1:894672-901060	273	4,00E-100	93.01

Chimpanzee (Pan troglodytes - Ptr) 48 chromosomes (22+X)	*CRLF3*	ENSPTRP00000015299	ENSPTRG00000008963	*CRLF3*	1	17:26256802-26298711	667	0.0	100.00
	ATAD5	ENSPTRP00000015300	ENSPTRG00000008964	*ATAD5*	1	17:26180838-26248737	2530	0.0	98.54
	ADAP2	ENSPTRP00000015304	ENSPTRG00000008966	*ADAP2*	1	17:26116861-26154177	588	0.0	99.21
	RNF135	ENSPTRP00000015306	ENSPTRG00000008967	*RNF135*	1	17:26079369-26104642	640	0.0	97.25
	NF1	ENSPTRP00000015312	ENSPTRG00000008970	*NF1*	1	17:25662187-25937192	4301	0.0	99.96
	UTP6	ENSPTRP00000015321	ENSPTRG00000008977	*UTP6*	1	17:25103021-25138303	777	0.0	99.23
	SUZ12	ENSPTRP00000015327	ENSPTRG00000008980	*SUZ12*	1	17:25002473-25065788	1036	0.0	99.70
	OMG	ENSPTRP00000015314	ENSPTRG00000008971	*OMG*	1	17:25736434-25737717	609	0.0	99.77
	LRRC37B	ENSPTRP00000015328	ENSPTRG00000034311	*ENSPTRG00000034311*	1	GL392684.1:11678-26241	951	0.0	97.38
		ENSPTRP00000061088	ENSPTRG00000041217	*ENSPTRG00000041217*	1	10:27597821-27599852	531	0.0	64.59
		ENSPTRP00000015329	ENSPTRG00000009573	*ENSPTRG00000009573*	1	GL392695.1:56411-91269	450	2,00E-158	49.09
		ENSPTRP00000015475	ENSPTRG00000009256	*ENSPTRG00000009256*	1	GL392694.1:45520-54108	330	2,00E-113	39.84
	EVI2A	ENSPTRP00000058537	ENSPTRG00000008973	*ENSPTRG00000008973*	1	17:25713549-25714256	368	4,00E-138	99.58
	EVI2B	ENSPTRP00000015315	ENSPTRG00000008972	*EVI2B*	2	17:25727151-25728455	616	0.0	98.39
	RAB11FIP4	ENSPTRP00000015317	ENSPTRG00000008974	*RAB11FIP4*	1	17:25500679-25603032	810	0.0	100.00
	TEFM	ENSPTRP00000015302	ENSPTRG00000008965	*TEFM*	1	17:26169195-26176581	554	0.0	99.44
	COPRS	ENSPTRP00000015319	ENSPTRG00000008976	*COPRS*	2	17:25146467-25154027	278	4,00E-102	95.70
Orangutan (Pongo abelii - Pab) 46 chromosomes (22+X) chromosome 2 divided in 2a e 2b)	*CRLF3*	ENSPPYP00000009152	ENSPPYG00000008142	*CRLF3*	1	17:25574996-25624139	667	0.0	100.00
	ATAD5	----------	----------	----------	----------	----------	-	-	-
	ADAP2	XP_002827275	Gene ID: 100442250	*ADAP2*	1	17:NC_012608.1	721	0.0	98.00
	RNF135	ENSPPYP00000009157	ENSPPYG00000008145	*RNF135*	1	17:25772856-25796806	632	0.0	96.06
	NF1	ENSPPYP00000009159	ENSPPYG00000008147	*NF1*	1	17:25942868-26211914	4293	0.0	99.86
	UTP6	ENSPPYP00000009166	ENSPPYG00000008154	*UTP6*	1	17:26707240-26772847	839	0.0	93.63
	SUZ12	XP_002827252	Gene ID: 100460330	*SUZ12*	1	17:NC_012608.1	649	0.0	99.51
	OMG	ENSPPYP00000009160	ENSPPYG00000008148	*DKFZP459F1351*	1	17:26136074-26137357	602	0.0	98.83
	LRRC37B	ENSPPYP00000009625	ENSPPYG00000008570	*ENSPPYG00000008570*	1	17:57868463-57901830	1136	0.0	89.77
		ENSPPYP00000002526	ENSPPYG00000002170	*ENSPPYG00000002170*	2	10:28292459-28297460	525	0.0	65.51
		ENSPPYP00000009158	ENSPPYG00000008146	*LRRC37B*	1	17:25886538-25894358	330	6,00E-115	44.50
		ENSPPYP00000009941	ENSPPYG00000008863	*ENSPPYG00000008863*	1	17_random:9615248-9621780	281	1,00E-95	42.41
		ENSPPYP00000009549	ENSPPYG00000008498	*ENSPPYG00000008498*	1	17:50407501-50414258	236	1,00E-77	41.40
	EVI2A	ENSPPYP00000009162	ENSPPYG00000008150	*ENSPPYG00000008150*	1	17:26159519-26160238	369	2,00E-138	97.92
	EVI2B	ENSPPYP00000009161	ENSPPYG00000008149	*EVI2B*	1	17:26145574-26146878	613	0.0	97.70
	RAB11FIP4	ENSPPYP00000009164	ENSPPYG00000008152	*RAB11FIP4*	1	17:26268215-26371054	765	0.0	99.82
	TEFM	ENSPPYP00000009154	ENSPPYG00000008143	*DKFZP459N1318*	1	17:25701008-25708035	545	0.0	97.78
	COPRS	ENSPPYP00000009165	ENSPPYG00000008153	*COPRS*	1	17:26695971-26702840	278	5,00E-102	96.24
Mouse (Rattus	*CRLF3*	NP_001162083	Gene ID: 54395	*Crlf3*	1	10:NC_005109.4	819	0.0	92.00

norvegicus - Rno) 44 chromosomes (20+X+Y)	*ATAD5*	ENSRNOP00000028975	ENSRNOG00000021903	*Atad5*	1	10:67427438-67473450	1719	0.0	68.86
	ADAP2	ENSRNOP00000053112	ENSRNOG00000037148	*Adap2*	1	10:67494984-67526423	468	3,00E-177	79.21
	RNF135	ENSRNOP00000005428	ENSRNOG00000004093	*Rhot1*	1	10:67532030-67558748	389	5,00E-143	59.91
	NF1	ENSRNOP00000050393	ENSRNOG00000013780	*Nf1*	2	10:66690133-66928166	4212	0.0	97.81
	UTP6	ENSRNOP00000019044	ENSRNOG00000014209	*Utp6*	1	10:67257161-67285617	753	0.0	82.91
	SUZ12	XP_008774000	Gene ID: 688041	*Suz12*	2	10:NC_005109.4	1172	0.0	87.00
	OMG	ENSRNOP00000018892	ENSRNOG00000014107	*Omg*	1	10:66846059-66847339	546	0.0	89.93
	LRRC37B	ENSRNOP00000042316	ENSRNOG00000042833	*Gm884*	1	10:91617260-91661555	273	4,00E-90	65.31
	EVI2A	ENSRNOP00000035290	ENSRNOG00000022764	*Evi2a*	1	10:66870624-66871286	226	5,00E-80	67.40
	EVI2B	ENSRNOP00000018898	ENSRNOG00000014125	*Evi2b*	1	10:66857378-66858598	296	6,00E-106	52.95
	RAB11FIP4	ENSRNOP00000018988	ENSRNOG00000014135	*Rab11fip4*	1	10:66942482-67047137	802	0.0	93.12
	TEFM	NP_001020797	Gene ID: 287554	*Tefm*	2	10:NC_005109.4	496	7,00E-175	68.00
	COPRS	ENSRNOP00000000123	ENSRNOG00000000111	*Coprs*	1	16:80826734-80831784	197	7,00E-69	73.81
Tarsier (Tarsius syrichta - Tsy) 46-80 chromosomes	*CRLF3*	ENSTSYP00000001673	ENSTSYG00000001807	*CRLF3*	1	GeneScaffold_6979:1858-14059	509	0.0	82.71
	ATAD5	ENSTSYP00000009057	ENSTSYG00000009871	*ATAD5*	1	GeneScaffold_6972:1092-43243	1767	0.0	72.07
	ADAP2	ENSTSYP00000019141.1	ENSTSYG00000032948	*ADAP2*	1	GeneScaffold KE948064.1: 146-29,881	249	3,00E-63	89.19
	*RNF135**	ENSTSYG00000034618	ENSTSYP00000032803.1	*RNF135*	1	GeneScaffold KE948064.1: 39,597-63,449	488	3,00E-135	86.74
	NF1	ENSTSYP00000008701	ENSTSYG00000009458	*NF1*	1	GeneScaffold_7882:63027-162075	2180	0.0	93.37
	UTP6	ENSTSYP00000002073	ENSTSYG00000002202	*UTP6*	1	GeneScaffold_1953:5132-31359	513	0.0	67.10
	SUZ12	ENSTSYP00000002783	ENSTSYG00000002995	*SUZ12*	1	GeneScaffold_7092:3386-59491	881	0.0	87.54
	OMG	ENSTSYP00000007966	ENSTSYG00000008689	*OMG*	1	scaffold_36978:4578-5864	583	0.0	96.50
	LRRC37B	----------	----------	----------	----------	----------	-	-	-
	EVI2A	ENSTSYP00000008702	ENSTSYG00000009480	*ENSTSYG00000009480*	1	GeneScaffold_7882:107567-109672	294	1,00E-107	74.24
	EVI2B	ENSTSYP00000012530	ENSTSYG00000013659	*EVI2B*	1	scaffold_119061:2831-3436	224	9,00E-77	78.16
	RAB11FIP4	ENSTSYP00000002970	ENSTSYG00000003234	*RAB11FIP4*	1	GeneScaffold_3344:2574-13968	328	7,00E-118	64.14
	TEFM	ENSTSYP00000009060	ENSTSYG00000009889	*TEFM*	1	GeneScaffold_6972:47361-52491	456	7,00E-173	84.24
	COPRS	XP_008046721	Gene ID: 103249911	*COPRS*	1	NW_007068165.1 (72286..79998)	262	2,00E-88	76.00

Red Cells: protein sequences excluded from the study because they are very different from the others.
* *Tarsius syrichata RNF135* and *Canis lupus LRRC37B* genes are absent because their sequence are mostly incomplete

Table S3. The best model of nucleotides and protein alignment.

Genes	Nucleotides models*	Amino acids models**
CRFL3	JC	JTT+I
ATAD5	JC	JTT+I+G
TEFM	JC	JTT+I
ADAP2	JC	JTT+I+G
RNF135	JC	JTT+G
NF1	JC	JTT+I
OMG	JC	JTT+I
EVI2B	JC	JTT+G
EVI2A	JC	FLU+G
RAB11FIP4	JC	JTT+G
COPRS	JC	JTT+I
UTP6	JC	JTT+G
SUZ12	JC	JTT+I
LRRC37B	JC	JTT+I+G

*calculated by JModelTest 2.1

**calculated by ProTest 2.4

Table S4. Positively selected sites in *RNF135* and amino acid differences between species.

RNF135 M8	4	5	**6****	8	12	30	34	47	**50****	51	56	**57***	58	**60***	61	62	69	72	75	76	**79****	94	95	**100***	103	**104***	105	106	107	108	109	113	115
Mice *(Mus musculus)*	V	C	S	N	?	G	Q	R	H	D	K	R	G	V	D	G	I	K	L	T	K	L	Q	V	G	S	E	P	E	?	?	?	?
Mouse *(Rattus norvegicus)*	A	C	P	T	?	G	W	Q	K	D	K	R	A	V	D	S	I	K	S	A	V	R	Q	L	G	P	E	P	A	?	?	?	?
Cattle (*Bos taurus*)	L	D	A	R	?	D	W	D	L	D	R	R	S	?	?	?	S	E	P	G	Q	R	L	L	L	S	?	?	?	?	?	E	T
Dog (*Canis lupus familiaris*)	P	D	P	P	?	G	W	D	K	G	G	C	A	P	P	?	T	A	A	E	Q	S	R	A	?	?	?	?	?	?	?	?	?
Marmoset (*Callithrix jacchus*)	L	G	L	P	P	G	W	R	E	S	R	A	A	G	R	?	T	E	A	Q	R	R	R	L	G	P	D	S	A	P	R	G	C
Orangutan (*Pongo abelii*)	L	G	P	?	S	G	W	H	E	G	R	G	V	R	R	?	T	Q	A	Q	Q	R	R	I	G	S	D	P	A	P	C	G	S
Gorilla (*Gorilla gorilla gorilla*)	?	?	?	?	?	?	?	?	?	?	?	?	?	?	?	?	?	?	?	?	?	R	R	I	G	S	D	P	A	H	C	G	S
Macaque (*Macaca mulatta*)	L	G	K	P	S	E	W	H	E	G	R	G	A	R	R	?	T	E	A	Q	R	R	R	L	G	S	N	S	A	A	C	G	S
Chimpanzee (*Pan troglodytes*)	L	G	L	P	S	G	W	H	E	A	R	G	A	R	R	?	T	Q	A	Q	Q	R	R	I	G	S	D	P	A	H	C	G	S
Human (*Homo sapiens*)	L	G	L	?	S	G	W	H	E	A	R	D	A	R	R	?	T	Q	A	Q	H	R	R	I	G	S	D	P	A	H	C	G	S

cont.

RNF135 M8	116	**117***	118	119	120	121	122	125	126	127	**128***	**129***	130	131	134	139	141	**143***	151	152	156	162	166	167	168	171	**175***	177
Mice *(Mus musculus)*	?	?	?	?	P	A	P	?	A	P	R	S	A	P	T	T	N	I	R	Q	D	T	N	L	G	Q	Q	T
Mouse *(Rattus norvegicus)*	?	?	?	?	P	V	P	L	C	T	P	T	P	P	T	T	Q	V	G	Q	I	T	S	L	A	L	L	I
Cattle (*Bos taurus*)	P	A	P	R	P	A	S	G	R	P	T	A	Q	L	E	S	E	D	E	R	I	S	L	L	E	P	V	V
Dog (*Canis lupus familiaris*)	?	?	?	?	?	?	?	?	?	?	?	?	?	?	?	I	E	G	E	Q	I	S	L	P	E	P	R	T
Marmoset (*Callithrix jacchus*)	P	?	?	?	?	A	P	R	R	R	G	S	L	P	E	I	E	G	E	R	I	S	L	S	E	P	L	I
Orangutan (*Pongo abelii*)	P	R	S	S	A	A	P	R	R	R	P	E	L	Q	A	I	E	G	E	H	I	N	L	S	E	P	L	I
Gorilla (*Gorilla gorilla gorilla*)	L	R	S	S	A	A	A	R	R	R	P	E	L	Q	A	I	E	G	E	H	I	N	L	S	E	P	L	I
Macaque (*Macaca mulatta*)	P	G	S	S	L	T	P	R	R	R	P	A	L	P	A	I	E	G	E	H	I	N	L	S	E	P	L	I
Chimpanzee (*Pan troglodytes*)	P	R	S	S	A	A	A	R	R	R	P	E	L	Q	A	V	E	G	E	H	I	N	L	S	E	P	L	I
Human (*Homo sapiens*)	S	L	S	S	A	A	A	R	R	R	P	E	L	Q	A	I	E	A	E	H	I	N	L	S	E	P	L	I

cont.

RNF135 M8	**179****	**181****	182	186	188	190	193	199	201	**202***	203	204	205	208	212	220	222	226	**227***	228	232	233	234	**235***	237	240	242
Mice *(Mus musculus)*	P	D	S	S	G	H	D	T	S	I	S	Q	K	Q	H	K	Q	P	G	R	R	E	R	V	?	?	E
Mouse *(Rattus norvegicus)*	H	D	S	S	E	Y	D	T	S	A	S	Q	K	Q	R	T	Q	T	G	N	K	K	Q	V	?	?	E
Cattle (*Bos taurus*)	A	T	L	D	V	L	A	S	S	P	P	E	Q	K	H	K	R	T	Q	K	E	E	Q	T	V	P	V
Dog (*Canis lupus familiaris*)	S	G	M	D	A	L	A	T	D	T	P	E	R	K	H	K	Q	T	W	K	E	E	H	L	V	Q	A
Marmoset (*Callithrix jacchus*)	G	T	F	S	V	L	A	T	D	A	A	A	G	K	H	K	R	S	W	K	E	A	P	T	R	L	A
Orangutan (*Pongo abelii*)	S	A	F	S	V	L	A	T	D	T	A	A	G	R	H	K	Q	T	R	K	E	A	Q	M	G	L	A
Gorilla (*Gorilla gorilla gorilla*)	G	A	F	S	V	L	A	T	D	T	A	A	G	R	H	K	Q	T	R	K	E	A	Q	M	G	L	A
Macaque (*Macaca mulatta*)	G	V	F	S	V	L	T	I	D	T	A	A	G	R	Q	K	R	A	R	K	E	A	Q	T	G	P	A
Chimpanzee (*Pan troglodytes*)	G	A	F	S	V	L	A	T	D	T	A	A	G	R	H	K	Q	T	R	K	E	A	Q	M	G	L	A
Human (*Homo sapiens*)	G	A	F	S	V	L	A	T	D	T	A	A	G	R	H	K	Q	T	W	K	E	A	Q	M	G	L	A

cont.

RNF135 M8	243	244	247	248	**255***	256	258	260	**263***	266	270	276	**286***	**288***	**290***	295	**297****	299	300	302	**305***	309	319	324	332	346	349
Mice *(Mus musculus)*	M	T	L	C	R	P	P	K	H	L	S	R	N	S	R	R	D	H	T	H	P	S	L	K	N	K	G
Mouse *(Rattus norvegicus)*	M	A	V	G	Y	P	S	K	Q	L	S	R	N	C	M	R	A	Q	P	H	S	S	F	K	N	K	K
Cattle (*Bos taurus*)	P	S	S	C	R	F	P	K	S	R	S	E	V	L	T	D	F	M	L	A	P	A	L	H	R	K	Q
Dog (*Canis lupus familiaris*)	P	S	S	H	C	P	P	R	Q	Q	S	R	E	C	V	H	W	Q	T	L	H	V	F	Q	H	R	Q
Marmoset (*Callithrix jacchus*)	Q	S	S	C	?	P	L	R	H	Q	N	K	R	F	T	P	R	Q	P	R	C	S	L	H	N	S	Q
Orangutan (*Pongo abelii*)	P	S	S	C	H	P	L	R	R	Q	H	K	K	S	T	H	R	Q	P	R	C	S	L	H	N	S	Q
Gorilla (*Gorilla gorilla gorilla*)	P	S	S	C	H	P	L	R	R	Q	R	K	K	S	T	H	R	Q	P	R	C	S	L	H	N	S	Q
Macaque (*Macaca mulatta*)	P	S	S	C	H	P	L	R	Q	Q	H	K	T	S	T	H	R	Q	P	R	C	S	L	H	N	S	Q
Chimpanzee (*Pan troglodytes*)	P	S	S	C	H	P	L	R	R	Q	H	K	K	S	T	H	R	Q	P	R	C	S	L	H	N	S	Q
Human (*Homo sapiens*)	P	S	S	C	H	P	L	R	R	Q	H	K	K	S	T	H	R	Q	P	R	C	S	L	H	N	S	Q

cont.

RNF135 M8	355	358	365	366	372	**380***	383	385	395	409	414	417	419	**431***	435	437	**439***	440	441	442	443	444
Mice *(Mus musculus)*	M	W	P	G	A	Q	L	E	S	L	E	S	S	S	Y	E	K	Q	L	N	T	?
Mouse *(Rattus norvegicus)*	M	W	P	S	A	S	P	E	L	P	E	S	S	T	Y	E	L	Q	I	Y	K	I
Cattle (*Bos taurus*)	T	W	A	S	C	G	R	S	E	P	P	V	V	D	S	T	R	K	M	N	V	?
Dog (*Canis lupus familiaris*)	K	Y	N	S	H	G	R	K	E	L	P	A	S	N	S	T	R	P	V	N	V	?
Marmoset (*Callithrix jacchus*)	T	C	T	N	H	G	R	G	E	L	T	A	S	H	Y	I	K	Q	V	K	V	?
Orangutan (*Pongo abelii*)	M	C	T	S	H	G	R	G	E	L	T	A	S	H	Y	I	K	Q	V	K	V	?
Gorilla (*Gorilla gorilla gorilla*)	M	C	T	S	H	G	R	G	E	L	T	A	S	H	Y	I	K	Q	V	K	V	?
Macaque (*Macaca mulatta*)	M	C	T	N	H	G	R	G	E	L	T	A	S	H	H	I	K	Q	V	K	V	?
Chimpanzee (*Pan troglodytes*)	M	C	T	S	H	G	R	G	E	L	T	A	S	H	Y	I	K	Q	V	K	V	?
Human (*Homo sapiens*)	M	C	T	S	H	G	R	G	E	L	T	A	S	H	Y	I	K	Q	V	K	V	?

Naive Empirical Bayes (NEB) p>95%*; p>99%** ; Bayes Empirical Bayes (BEB) p>95%#; p>99%##

Table S5. Positively selected sites in *UTP6* and amino acid differences between species.

UTP6 M8	73	204	345	357	371	378	382	383	384	412	416	423	425	544	585
Mice *(Mus musculus)*	A	A	V	L	V	D	R	Q	D	Q	D	E	R	S	L
Mouse *(Rattus norvegicus)*	A	A	V	R	V	D	R	Q	D	L	D	G	H	T	M
Cattle (*Bos taurus*)	A	N	R	V	L	K	H	R	Q	E	T	S	G	N	Q
Dog (*Canis lupus familiaris*)	T	K	Q	M	F	D	H	H	D	Q	N	A	C	V	M
Tarsier (*Tarsius syrichta*)	L	S	G	T	V	?	?	?	?	?	?	?	?	T	A
Gibon (*Nomascus leucogenys*)	T	S	G	T	C	V	C	H	N	R	E	A	L	A	A
Marmoset (*Callithrix jacchus*)	K	S	G	T	F	K	S	H	K	Q	D	A	Q	A	A
Orangutan (*Pongo abelii*)	T	S	G	T	C	V	C	R	N	R	E	A	L	A	A
Gorilla (*Gorilla gorilla gorilla*)	T	S	G	T	C	V	C	H	N	Q	E	A	L	A	A
Macaque (*Macaca mulatta*)	T	S	G	T	C	V	C	H	K	R	E	A	L	T	A
Chimpanzee (*Pan troglodytes*)	T	S	G	T	C	V	R	Y	N	Q	E	A	L	?	?
Human (*Homo sapiens*)	T	S	G	T	C	V	C	Y	N	Q	E	A	L	A	A

Naive Empirical Bayes (NEB) p>95%*; p>99%** ; Bayes Empirical Bayes (BEB) p>95%#; p>99%##

Table S6. Positively selected sites in *LRRC37B* and amino acid differences between species.

LRRC37B M2	3	19	372	386	397	402	406	923	959
Mice *(Mus musculus)*	V	P	N	L	I	E	E	G	C
Mouse *(Rattus norvegicus)*	?	?	S	F	I	E	E	A	Y
Cattle (*Bos taurus*)	V	W	K	P	S	E	S	A	Y
Gibon (*Nomascus leucogenys*)	W	Y	N	N	A	E	E	M	L
Marmoset (*Callithrix jacchus*)	?	?	S	V	K	E	Q	F	S
Orangutan (*Pongo abelii*)	?	S	N	I	S	R	A	?	?
Gorilla (*Gorilla gorilla gorilla*)	W	S	N	I	F	M	A	?	?
Macaque (*Macaca mulatta*)	P	T	F	A	L	T	A	T	R
Chimpanzee (*Pan troglodytes*)	W	C	S	I	S	T	A	I	H
Human (*Homo sapiens*)	W	S	N	I	S	R	D	M	N

LRRC37B M8	3	19	82	158	234	289	324	340	371	372	375	386	397	402	406	645	668	834	923	959
Mice *(Mus musculus)*	W	Y	L	K	S	L	M	Q	K	N	A	N	A	E	E	R	H	S	M	L
Mouse *(Rattus norvegicus)*	?	?	?	Y	Q	L	N	I	D	S	N	F	I	E	E	F	E	T	A	Y
Cattle (*Bos taurus*)	V	W	D	G	H	E	Q	S	T	K	E	P	S	E	S	R	F	S	A	Y
Gibon (*Nomascus leucogenys*)	W	S	S	W	S	L	M	Q	K	N	T	I	F	M	A	?	?	?	?	?
Marmoset (*Callithrix jacchus*)	?	?	?	R	Q	R	Q	I	S	S	L	V	K	E	Q	I	T	S	F	S
Orangutan (*Pongo abelii*)	W	C	S	K	G	L	V	Q	K	S	T	I	S	T	A	H	L	S	I	H
Gorilla (*Gorilla gorilla gorilla*)	W	S	S	W	S	L	M	Q	K	N	T	I	S	R	D	Q	L	S	M	N
Macaque (*Macaca mulatta*)	V	P	M	Y	Q	L	H	I	G	N	N	L	I	E	E	Y	A	V	G	C
Chimpanzee (*Pan troglodytes*)	?	S	S	W	S	L	M	Q	K	N	T	I	S	R	A	Q	L	?	?	?
Human (*Homo sapiens*)	P	T	S	K	S	F	M	Q	K	F	V	A	L	T	A	R	F	S	T	R

Naive Empirical Bayes (NEB) p>95%*; p>99%** ; Bayes Empirical Bayes (BEB) p>95%#; p>99%##

Table S7. Positively selected sites in *EVI2B* and amino acid differences between species.

EVI2B M8	30	37	41	42	43	56	65	76	104	106	113	120	135	143	166	171	185	186	194	197	199	202	207	281	320	360	399	400	422	428	441	446
Mice *(Mus musculus)*	K	V	M	S	H	L	I	V	Y	?	M	Q	H	T	T	P	R	S	V	N	Q	Q	Y	P	A	S	I	I	N	L	S	N
Mouse *(Rattus norvegicus)*	H	P	A	V	H	L	I	T	?	?	I	G	H	K	L	R	P	P	P	G	G	H	H	P	M	E	I	N	I	R	L	M
Cattle (*Bos taurus*)	R	F	M	P	Y	S	L	I	Y	V	I	G	Y	?	S	I	R	K	F	T	N	K	A	V	A	G	S	K	N	N	F	N
Dog (*Canis lupus familiaris*)	K	L	R	Q	H	M	V	D	S	P	T	Q	P	?	S	P	R	N	T	N	N	H	S	P	A	S	F	K	N	N	S	S
Tarsier (*Tarsius syrichta*)	K	F	M	P	?	?	?	?	?	?	?	?	?	?	?	?	?	?	?	?	?	?	?	T	A	N	I	R	V	I	S	D
Gibon (*Nomascus leucogenys*)	K	F	M	S	H	L	T	V	Y	?	M	Q	H	T	T	P	K	S	T	N	Q	Q	Y	P	V	S	I	I	N	L	P	N
Marmoset (*Callithrix jacchus*)	K	F	T	S	Y	M	T	A	S	?	T	Q	H	T	P	P	R	S	A	N	R	Q	N	P	V	S	T	I	N	L	S	N
Orangutan (*Pongo abelii*)	K	F	T	S	H	L	T	V	Y	?	M	Q	H	P	T	P	R	S	T	N	Q	Q	Y	P	V	S	I	I	N	L	S	N
Gorilla (*Gorilla gorilla gorilla*)	K	L	M	S	H	L	T	V	Y	?	I	Q	H	K	T	P	R	S	T	N	Q	H	Y	P	V	S	I	M	N	L	S	N
Macaque (*Macaca mulatta*)	K	V	M	S	H	L	I	V	Y	?	M	Q	H	T	T	P	R	S	V	N	Q	Q	Y	P	A	S	I	I	N	L	S	N
Chimpanzee (*Pan troglodytes*)	K	F	M	S	H	L	T	V	Y	?	I	Q	H	K	T	P	R	S	T	N	Q	Q	Y	P	V	S	I	I	N	L	C	N
Human (*Homo sapiens*)	K	F	M	S	Q	L	T	V	Y	?	I	Q	R	K	T	P	R	S	T	N	Q	Q	Y	P	V	S	I	I	N	L	S	N

Naive Empirical Bayes (NEB) p>95%*; p>99%** ; Bayes Empirical Bayes (BEB) p>95%#; p>99%##

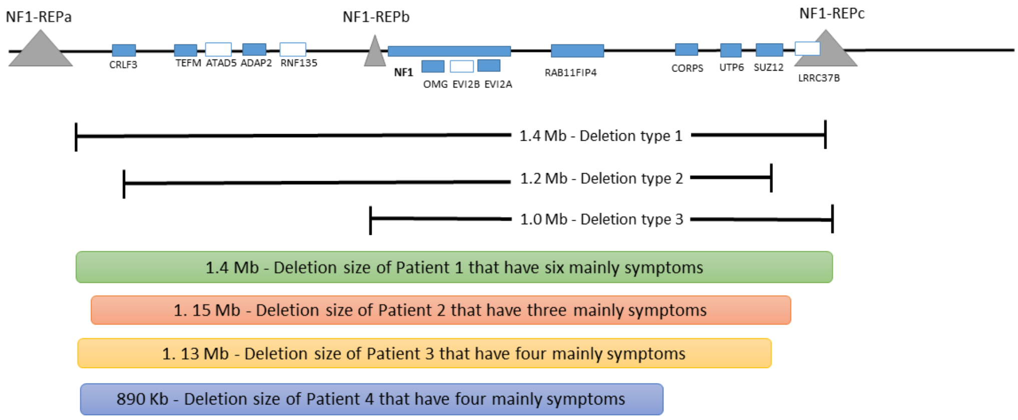

Supplementary Figure 1. Representation of existing microdeletions in patients with neurofibromatosis (type 1, 2 and 3 deletions) and deletions found in the previously analyzed patients [18].Gray triangles represent the breakpoint regions NF1REPa, NF1REPb and NF1REPc. Blue rectangles indicate functional genes that are under purifying selection, empty rectangles indicate the functional genes under positive selection, colored rectangles represent the sizes of deletions found in the patients recruited in this study.

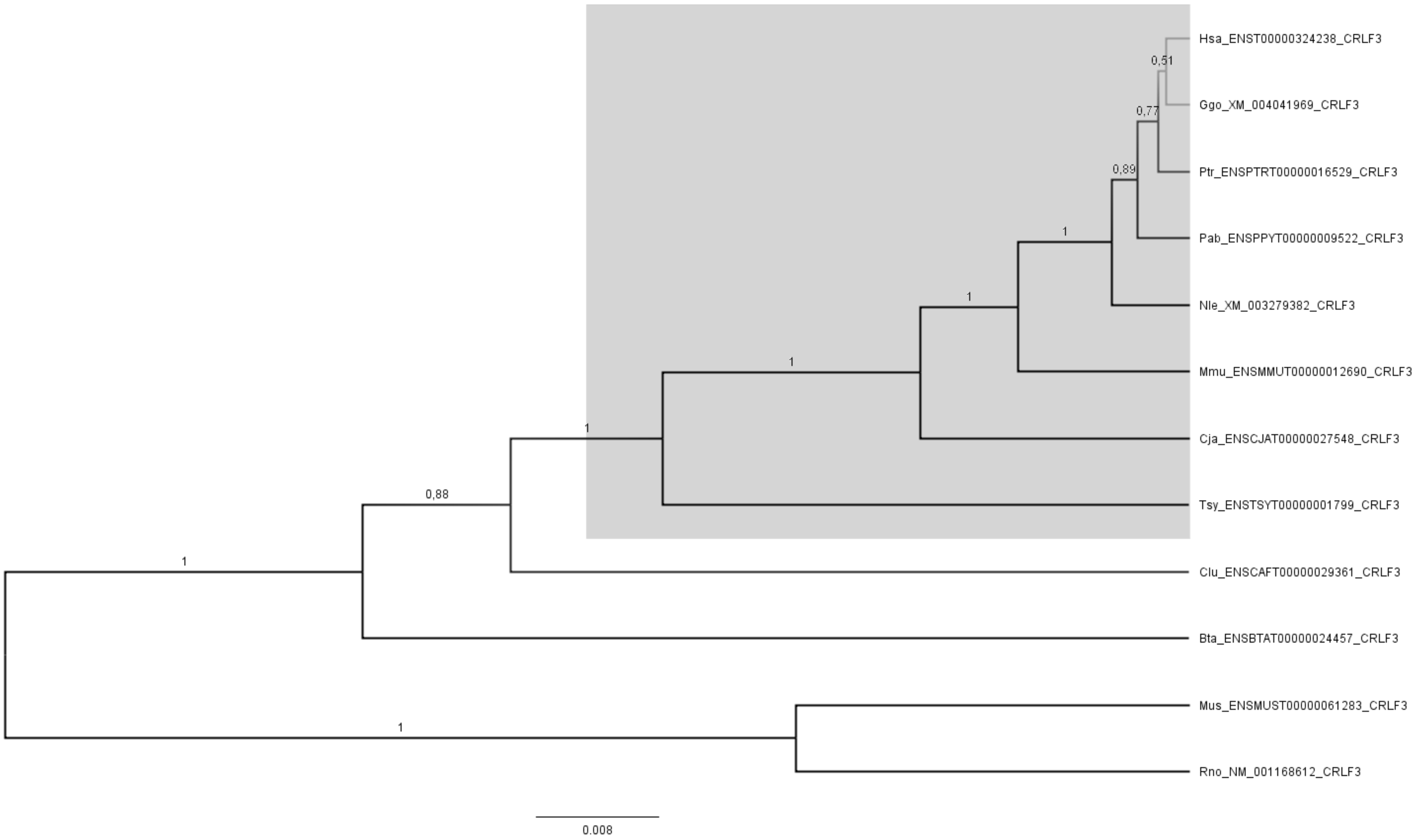

Supplementary Figure 2. Phylogenetic tree of *CRLF3* gene reconstructed based on nucleotides sequences.

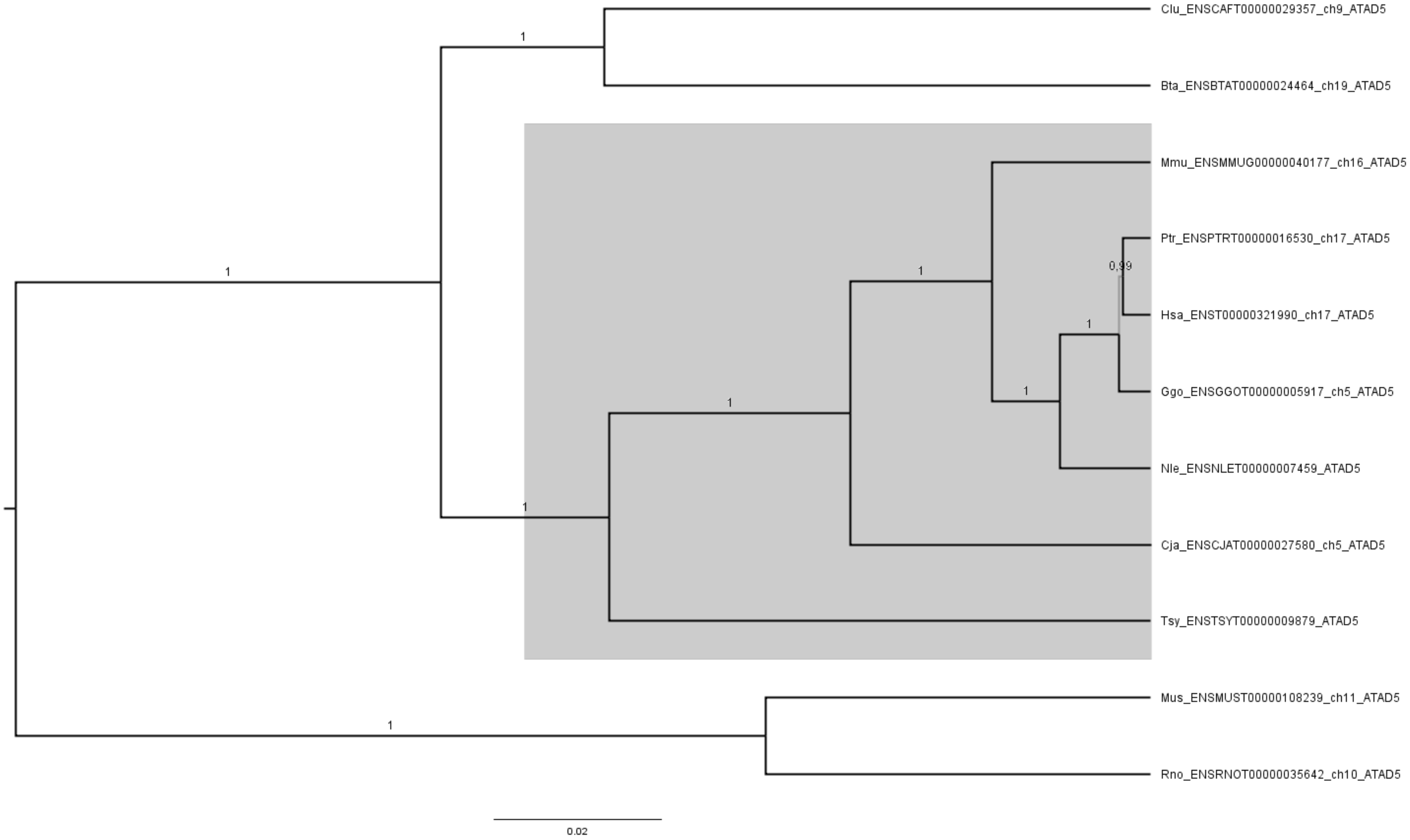

Supplementary Figure 3. Phylogenetic tree of *ATAD5* gene reconstructed based on nucleotides sequences.

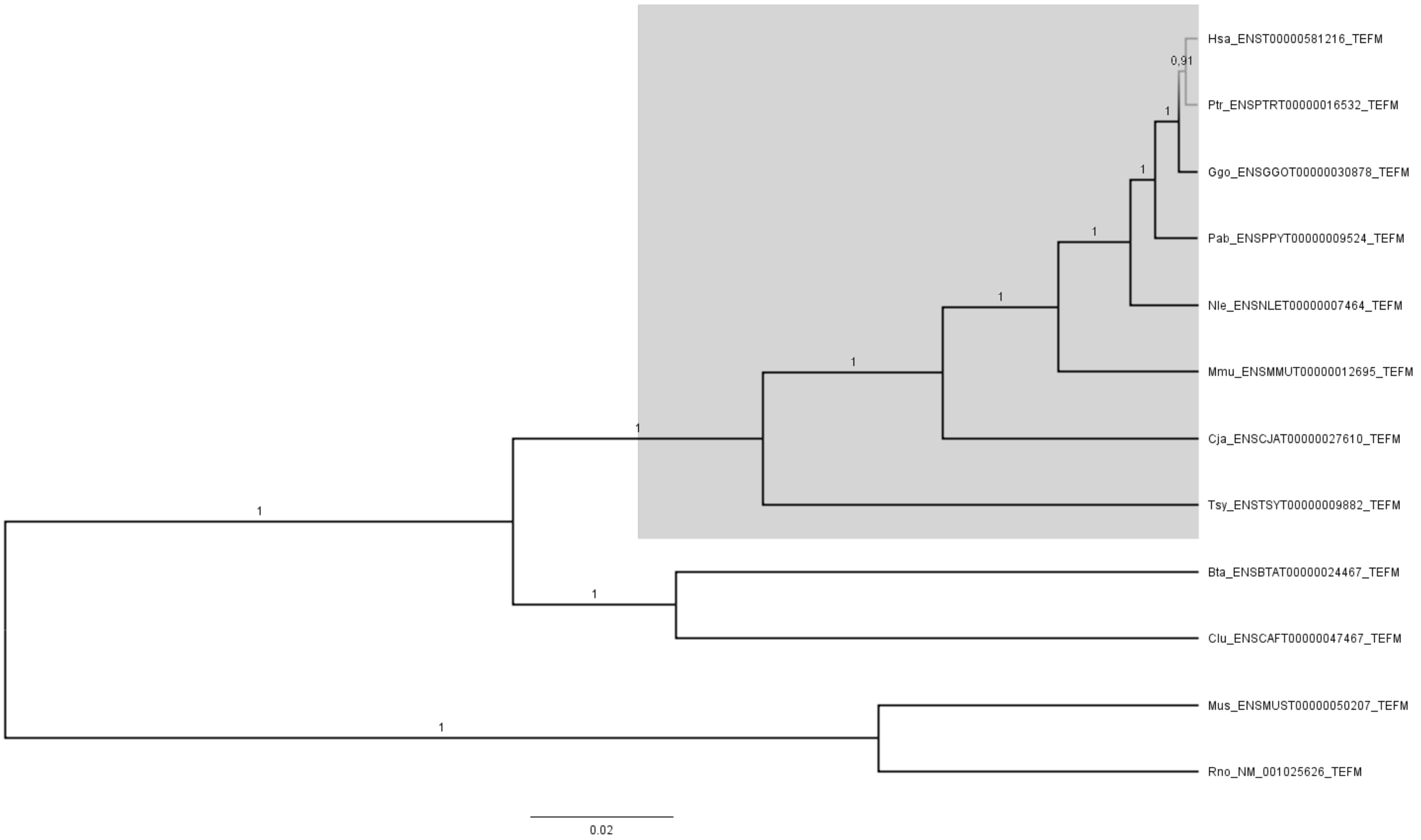

Supplementary Figure 4. Phylogenetic tree of *TEFM* gene reconstructed based on nucleotides sequences.

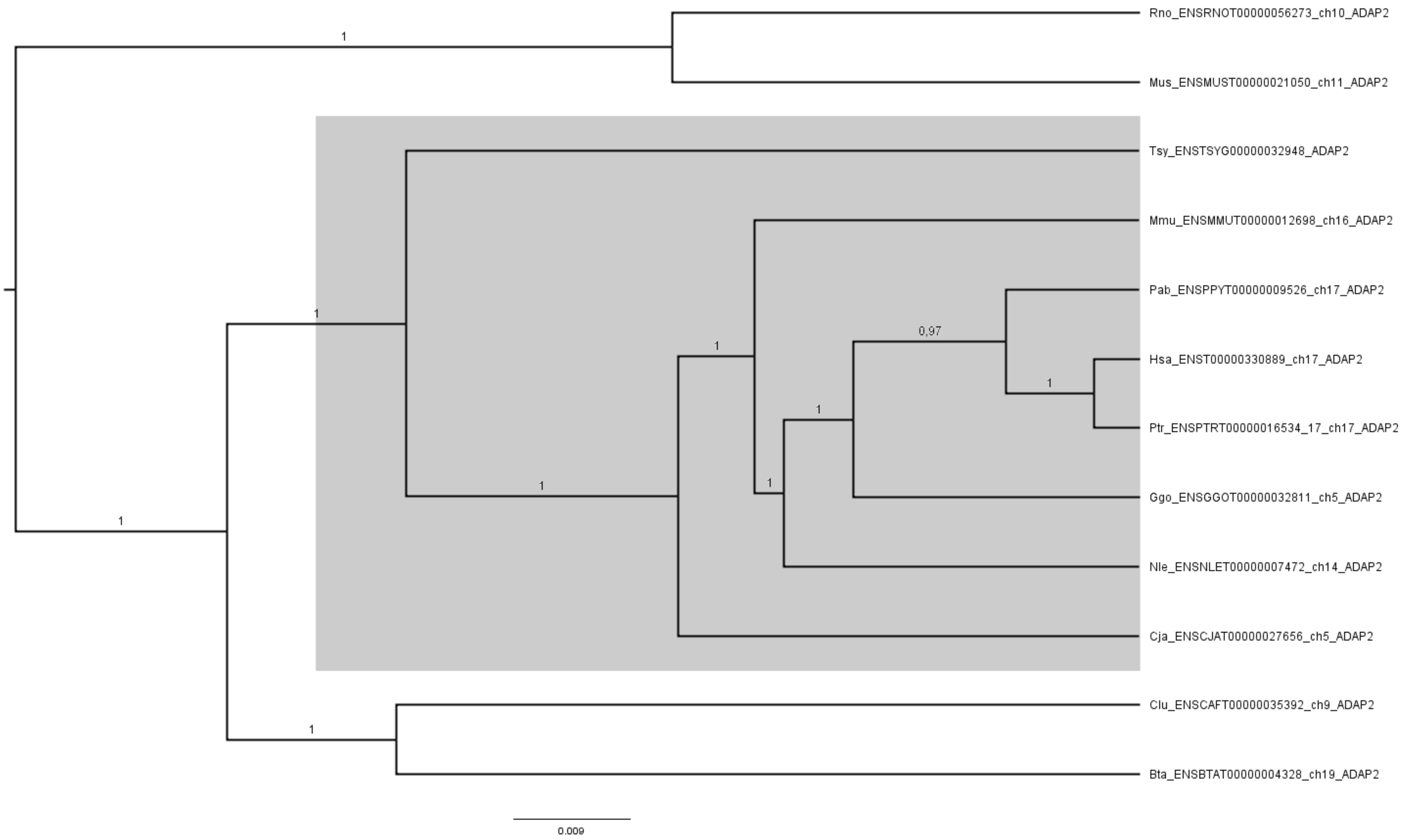

Supplementary Figure 5. Phylogenetic tree of *ADAP2* gene reconstructed based on nucleotides sequences.

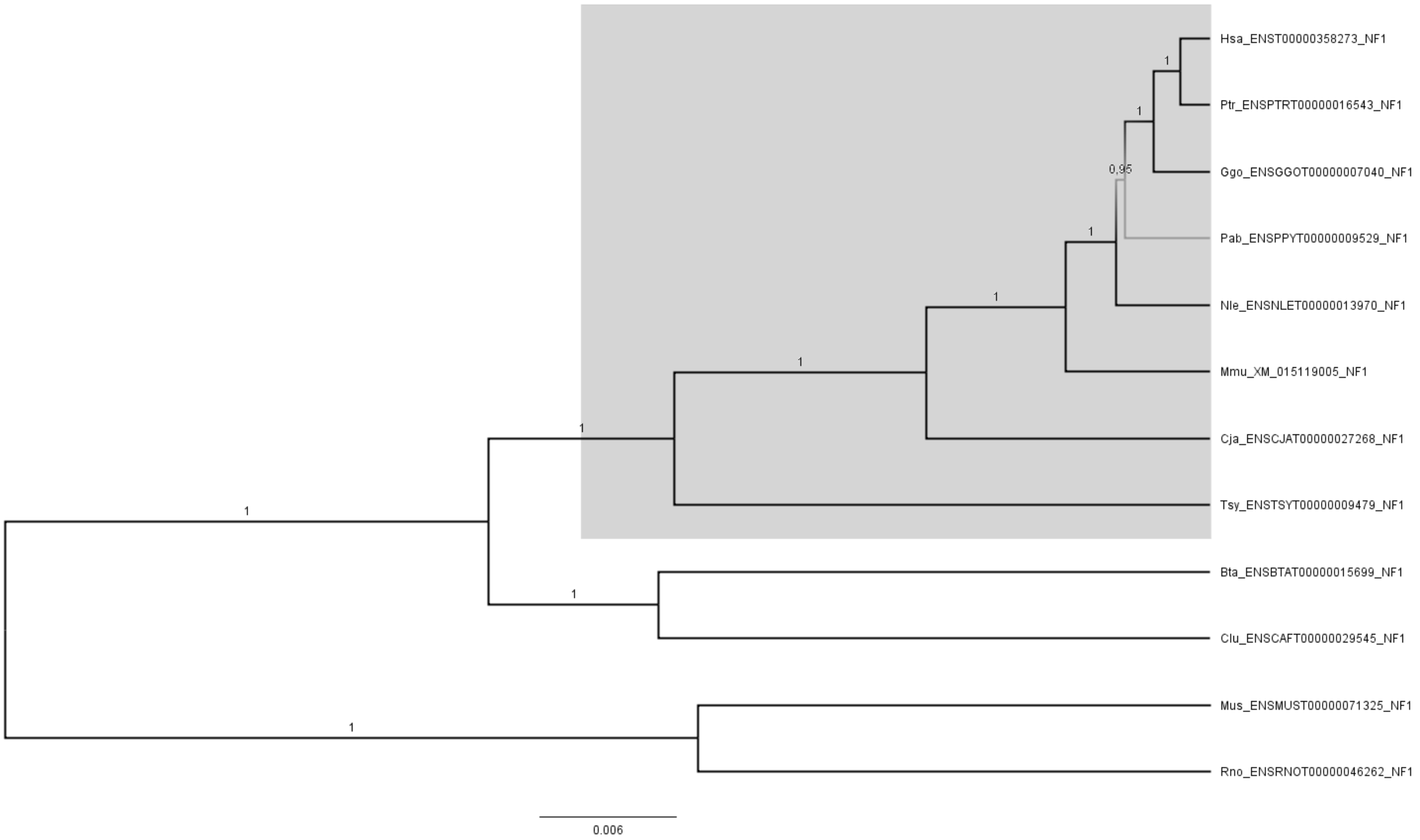

Supplementary Figure 6. Phylogenetic tree of *NF1* gene reconstructed based on nucleotides sequences.

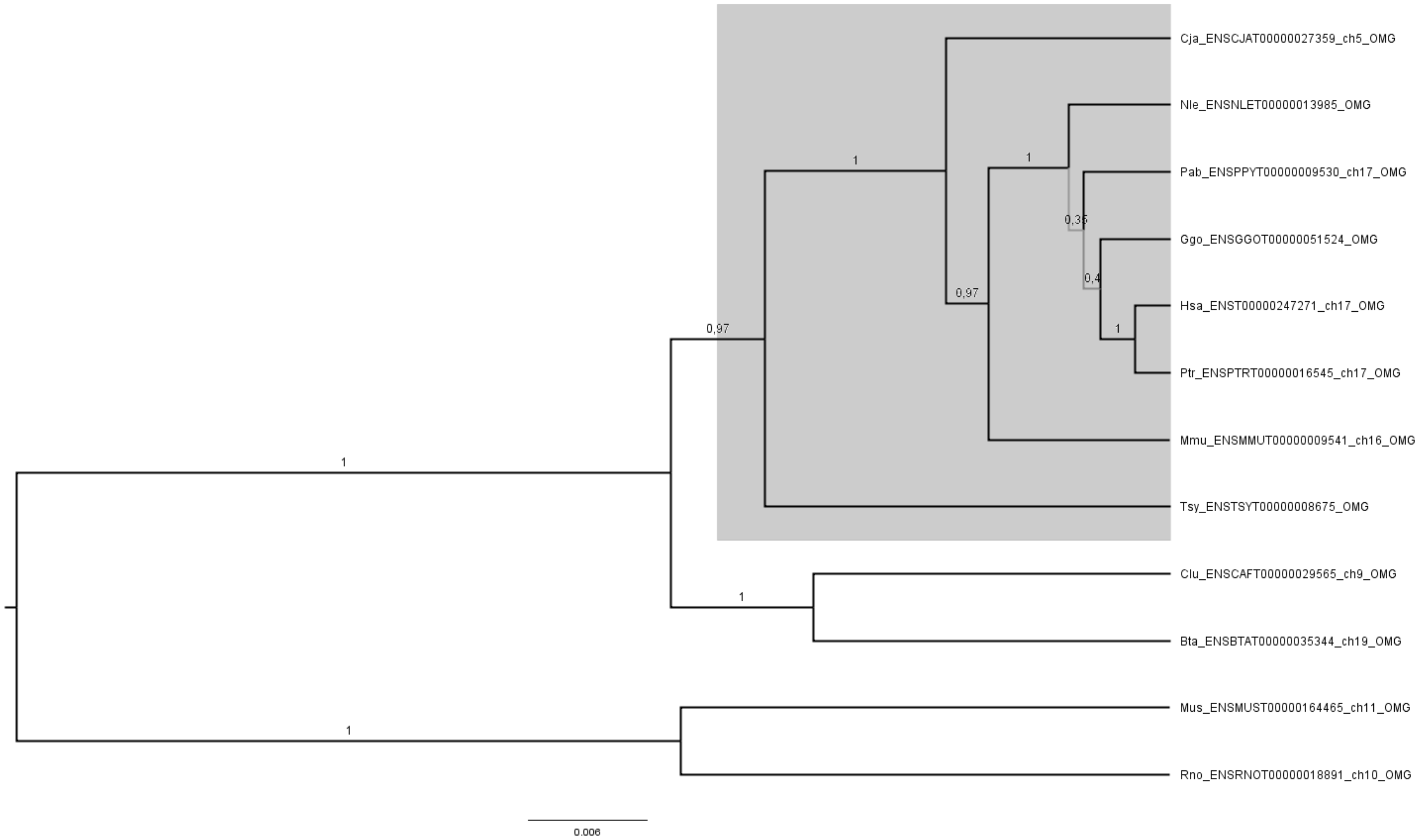

Supplementary Figure 7. Phylogenetic tree of *OMG* gene reconstructed based on nucleotides sequences.

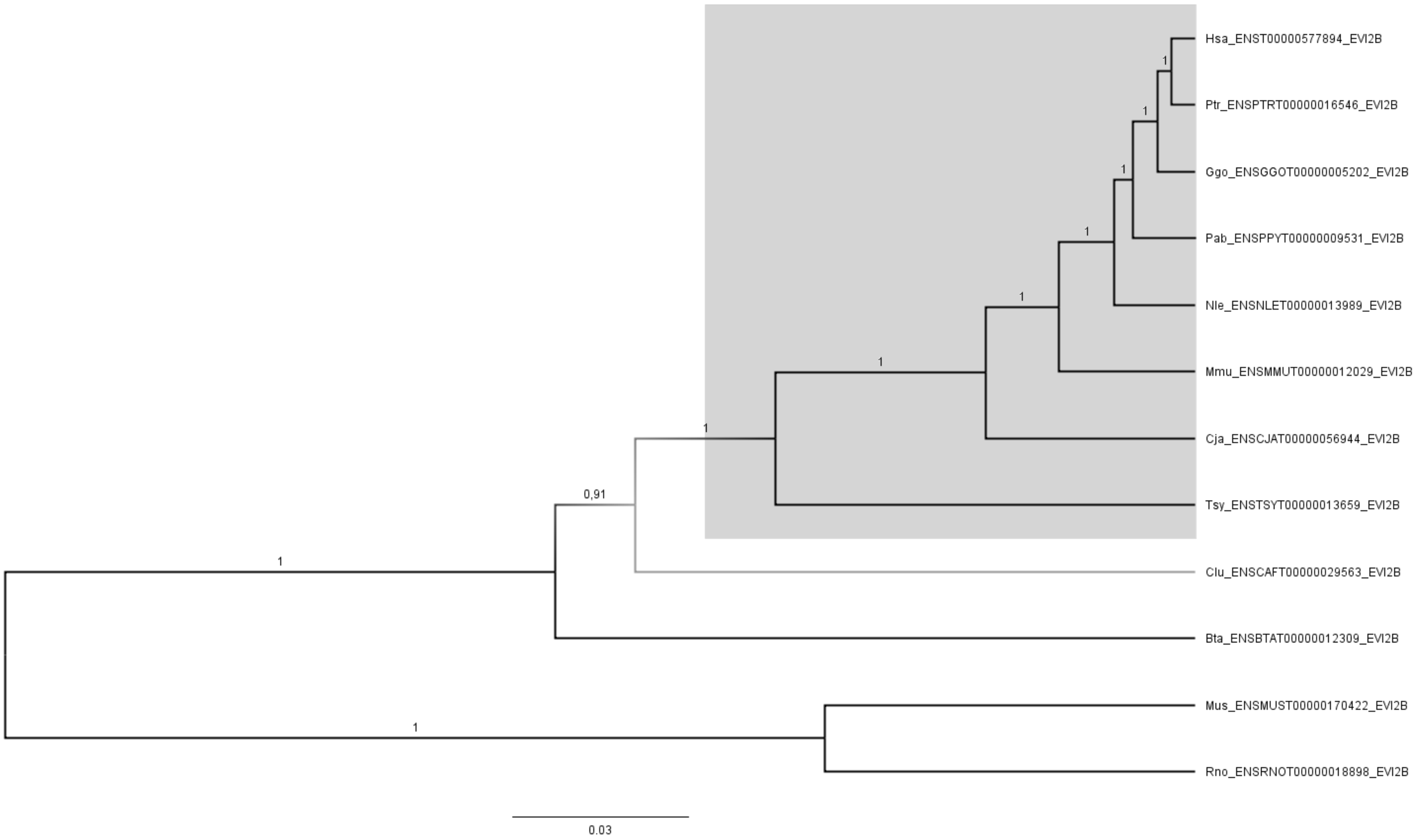

Supplementary Figure 8. Phylogenetic tree of *EVI2B* gene reconstructed based on nucleotides sequences.

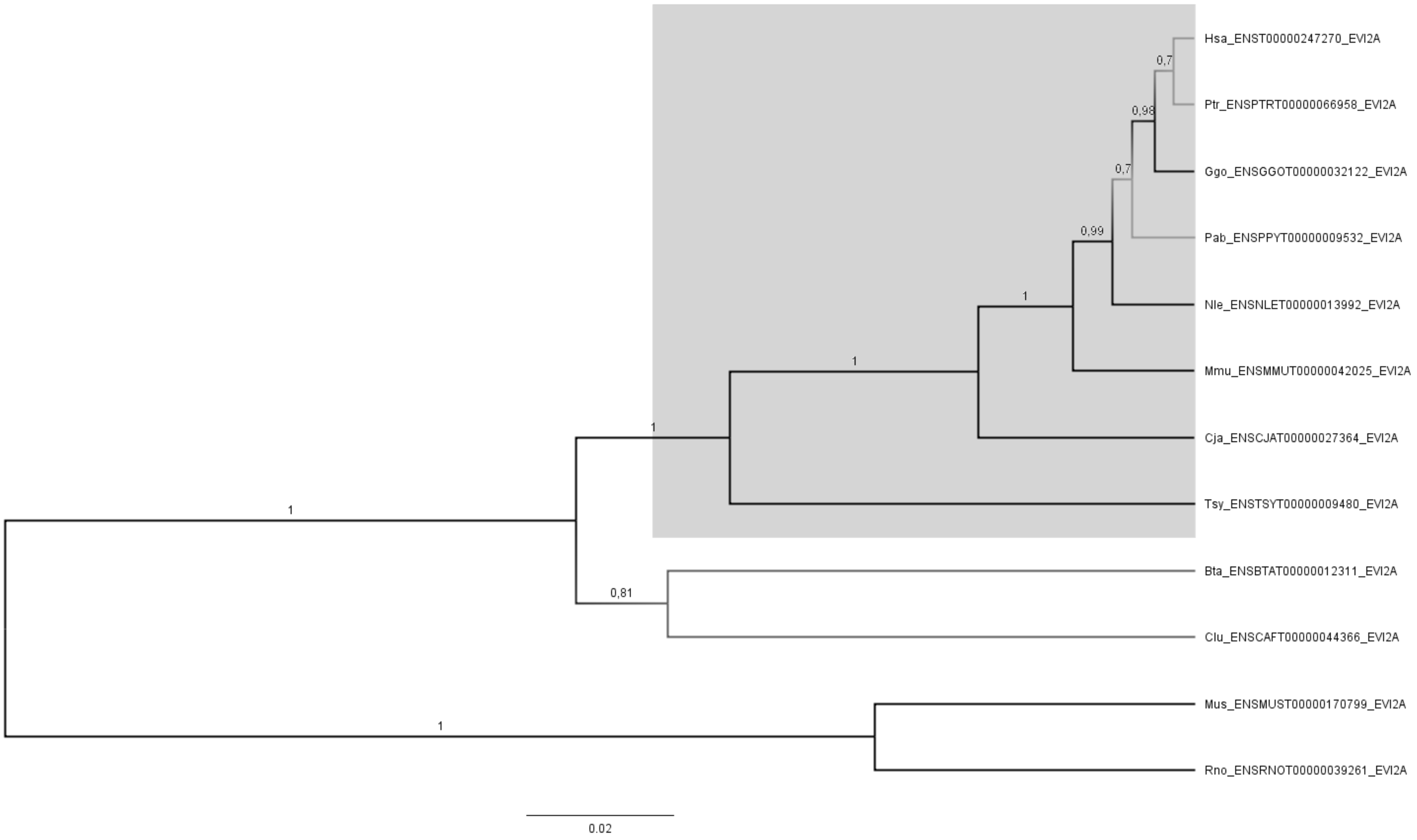

Supplementary Figure 9. Phylogenetic tree of *EVI2A* gene reconstructed based on nucleotides sequences.

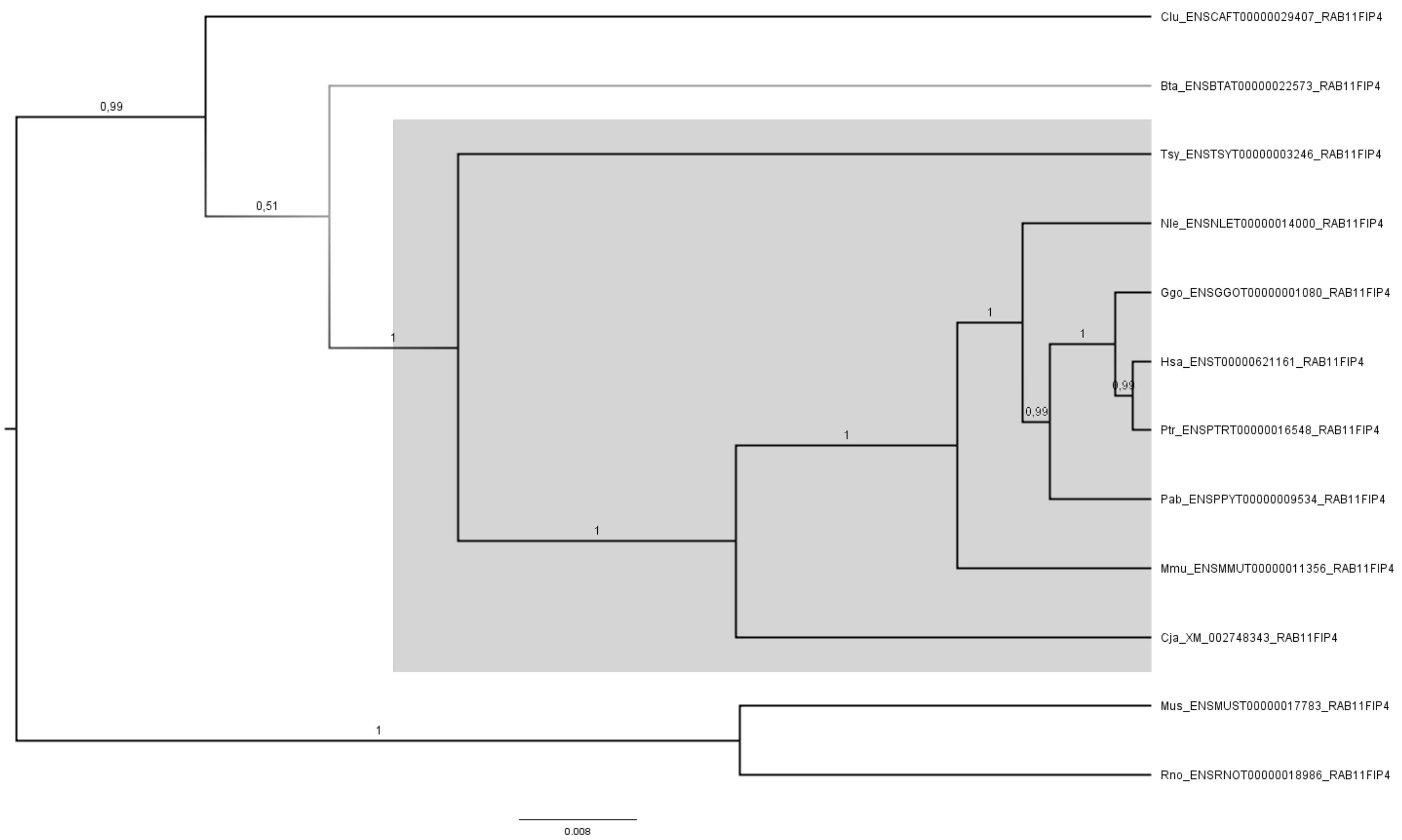

Supplementary Figure 10. Phylogenetic tree of *RAB11FIP4* gene reconstructed based on nucleotides sequences.

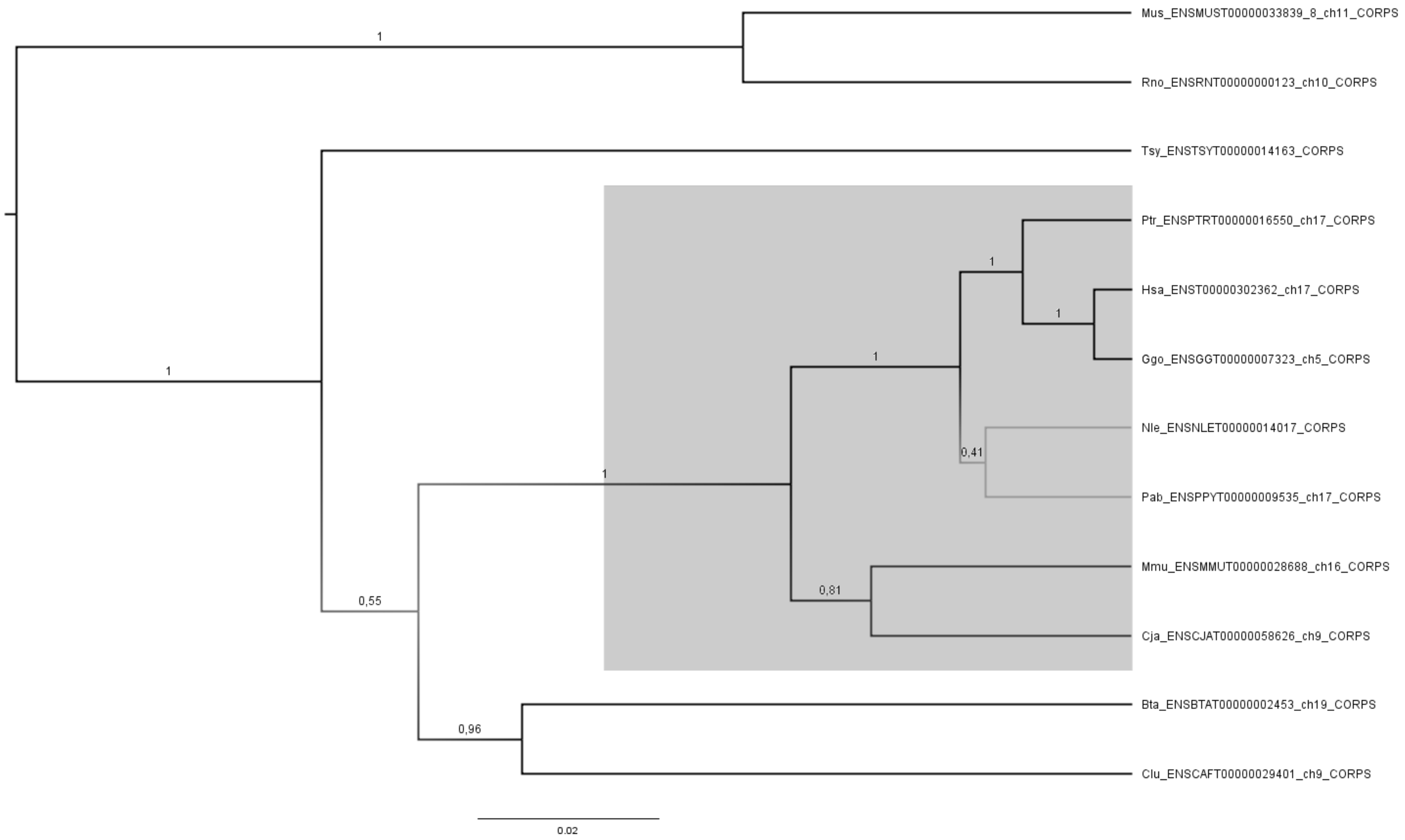

Supplementary Figure 11. Phylogenetic tree of *COPRS* gene reconstructed based on nucleotides sequences.

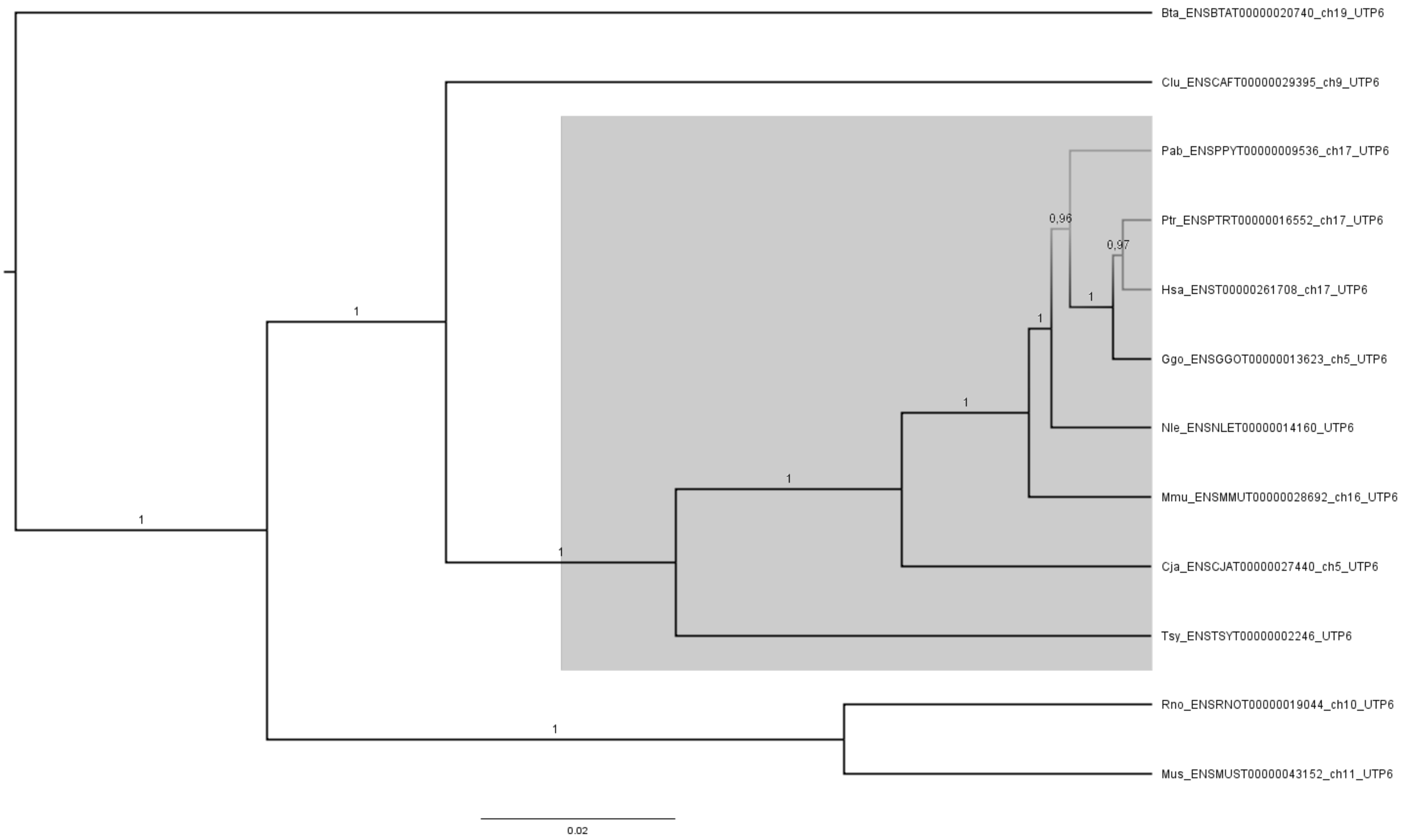

Supplementary Figure 12. Phylogenetic tree of *UTP6* gene reconstructed based on nucleotides sequences.

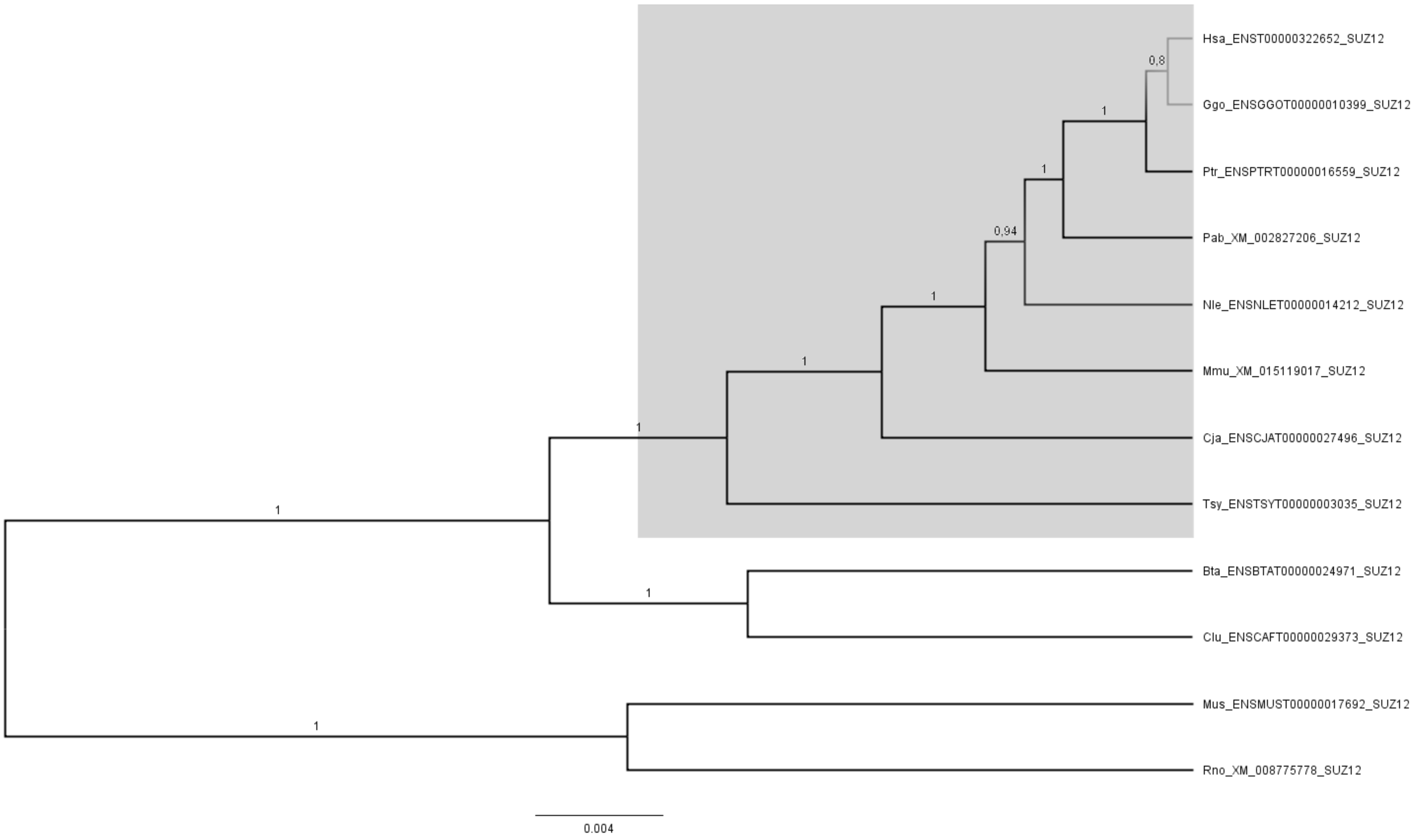

Supplementary Figure 13. Phylogenetic tree of *SUZ12* gene reconstructed based on nucleotides sequences.

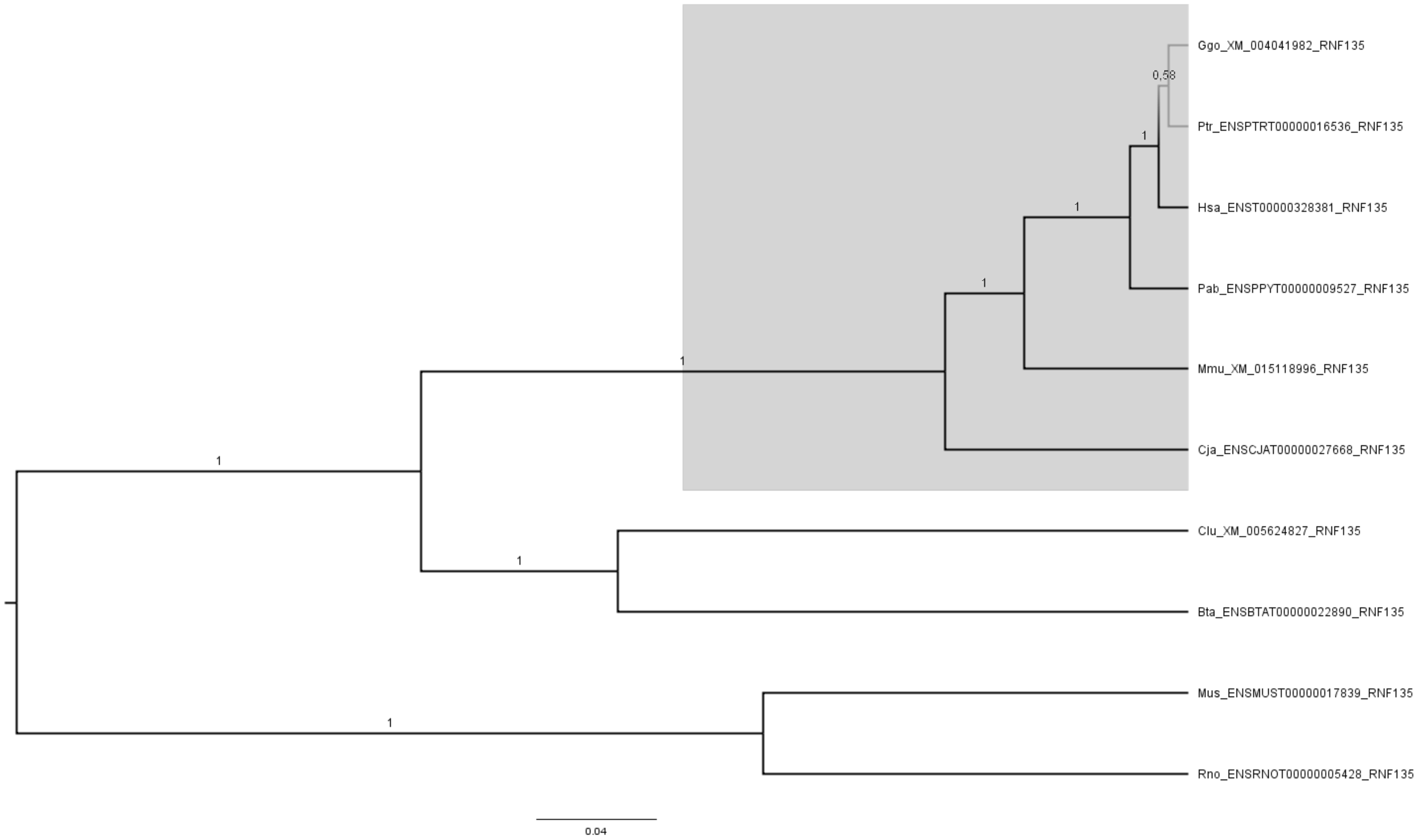

Supplementary Figure 14. Phylogenetic tree of *RNF135* gene reconstructed based on amino acid sequences.

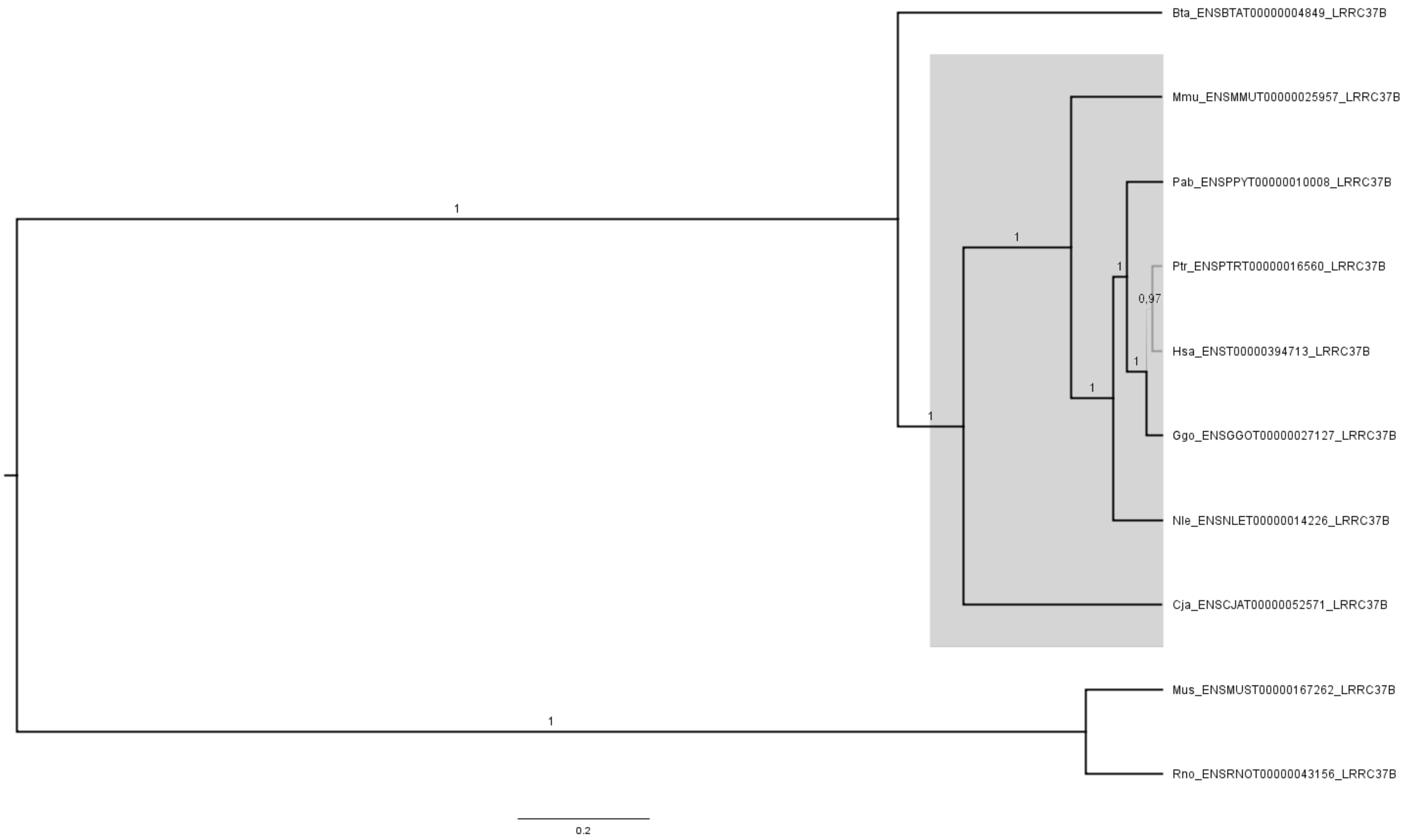

Supplementary Figure 15. Phylogenetic tree of *LRRC37B* gene reconstructed based on amino acid sequences.
